# Exploring the In Vivo Effects of Amyloid‐Beta Oligomers on Neuronal Activity and Neurotransmitter Release

**DOI:** 10.1002/alz70855_101022

**Published:** 2025-12-23

**Authors:** Vincent Hervé, Obai BinB KaAli, Habib Benali, Jonathan Brouillette

**Affiliations:** ^1^ Université de Montréal, Montréal, QC, Canada; ^2^ Concordia University, Montreal, QC, Canada; ^3^ PERFORM Centre, University of Concordia, Montreal, QC, Canada

## Abstract

**Background:**

One of the main neuropathological hallmarks of Alzheimer's disease (AD) is the accumulation of amyloid‐beta oligomers (Aβo), which begins in the brain approximately 15 years prior to the onset of clinical symptoms. Aβo‐induced neuronal hyperactivity has emerged as an early functional characteristic of AD, contributing to synaptic deficits, memory impairment, and neurodegeneration. Several studies suggest that Aβo reduce the inhibitory activity of the GABAergic system, leading to excessive activation of the glutamatergic system. The aim of this study is to investigate the impact of Aβo on neuronal activity and neurotransmitter release within the same animal model.

**Method:**

A microdialysis probe is implanted in the right hippocampus of a rat model to deliver Aβ oligomers over five consecutive days and to collect interstitial fluid (ISF) samples before, during, and after the injections. In addition, five electrodes are implanted within the hippocampi and default mode network of the rats to record neuronal activity.

**Result:**

Firstly, we demonstrated through immunohistochemistry that our Aβo injections are successfully delivered into the hippocampus. Our initial results demonstrate the ability to record local field potential (LFP) signals in five distinct brain areas and to detect several neurotransmitters (glutamate, GABA, serine, taurine, glutamine, and glycine) in ISF samples. Chronic Aβo injections resulted in a clear time‐dependent increase in delta power and a progressive decrease in higher frequency bands (theta, alpha, beta, gamma) in the injected hippocampus. In the non‐injected (left) hippocampus, stable trends were observed initially, with a late onset increase in delta power and reduction in higher frequency power by day 5 in the Aβo group. Preliminary results on the quantification of neurotransmitters, including glutamate and GABA, are currently being analyzed but have already been detected by mass spectrometry in the ISF fractions.

**Conclusion:**

This study will provide novel insights into the relationship between Aβo‐induced changes in neuronal activity and neurotransmitter release, contributing to a better understanding of the neurodegenerative processes involved in early AD.

